# Differential molecular mechanisms of bortezomib sensitization to rhTRAIL in non-small cell lung cancer cell lines

**DOI:** 10.37349/etat.2025.1002342

**Published:** 2025-10-28

**Authors:** Paweł Kochany, Janet H. Stegehuis, Leonie H.A.M. de Wilt, Gerrit Jansen, Steven de Jong, Godefridus J. Peters, Frank A.E. Kruyt

**Affiliations:** Università degli Studi della Campania “Luigi Vanvitelli”, Italy; ^1^Department of Biochemistry, Medical University of Gdansk, 80-211 Gdansk, Poland; ^2^Department of Medical Oncology, University Medical Center Groningen, University of Groningen, 9700 RB Groningen, The Netherlands; ^3^Department of Medical Oncology, Amsterdam University Medical Centers, Vrije Universiteit Amsterdam, 1007 MB Amsterdam, The Netherlands; ^4^Department of Rheumatology, Amsterdam University Medical Centers, Vrije Universiteit Amsterdam, 1081 HV Amsterdam, The Netherlands

**Keywords:** non-small cell lung cancer, TNF-related apoptosis-inducing ligand (TRAIL), bortezomib, apoptosis, rhTRAIL-resistance

## Abstract

**Aim:**

TNF-related apoptosis-inducing ligand (TRAIL) is a promising targeted anti-cancer agent for several types of cancer, including non-small cell lung cancer (NSCLC). The proteasome inhibitor bortezomib can further potentiate rhTRAIL-induced apoptosis in NSCLC cells. Here, the mechanisms underlying this sensitization were examined in TRAIL-sensitive H460 and TRAIL-resistant A549 and SW1573 NSCLC cells.

**Methods:**

NSCLC cell lines were treated with rhTRAIL and bortezomib, and apoptosis was assessed through caspase activation assays, western blotting, and gene silencing of key apoptotic regulators, including Bid, XIAP, and cFLIP. Clonogenic assays were performed to evaluate long-term tumor growth suppression.

**Results:**

Bortezomib sensitization mechanisms varied across NSCLC cell lines. Combined rhTRAIL/bortezomib treatment enhanced apoptosis across all cell lines. In TRAIL-sensitive H460 cells, rapid caspase activation was observed, with both extrinsic and intrinsic apoptotic pathways contributing to cell death. Sensitization in H460 cells was predominantly mediated via the caspase-8/Bid amplification loop. In A549 cells, the bortezomib sensitizing effect also relied on the caspase-8/Bid amplification loop. Additionally, the inhibition of Bid and XIAP emphasized the critical role of mitochondrial pathways in apoptosis. In SW1573 cells, limited caspase cleavage was detected, with distinct cleavage patterns suggesting cell-specific apoptotic mechanisms. In this cell line, bortezomib primarily enhanced the extrinsic apoptotic pathway, with XIAP depression further increasing apoptosis. Silencing cFLIP, a caspase-8 inhibitor, significantly improved rhTRAIL sensitivity, emphasizing the critical role of caspase-8 activation in overcoming resistance in SW1573. The clonogenic assay demonstrated that bortezomib combined with rhTRAIL significantly suppressed tumor growth, especially in resistant cell lines.

**Conclusions:**

This study underscores bortezomib’s ability to differentially enhance rhTRAIL-induced apoptosis by targeting multiple apoptotic regulators. The variety of effects that bortezomib can exert to enhance rhTRAIL-induced apoptosis makes it a very powerful combination for the treatment of NSCLC and various other types of cancer cells.

## Introduction

Lung cancer is the second most often diagnosed cancer type worldwide, representing 11.4% of all cancers [1]. Non-small cell lung cancer (NSCLC) represents 85% of the patients diagnosed with lung cancer and is the leading cause of cancer-related deaths [2]. Depending on the disease stage and driver gene mutations, current treatments consist of surgery, radiation, chemotherapy, targeted pharmacological therapy and immunotherapy, all active in subsets of patients. Although efficacy has been shown in subgroups of patients, for the majority of patients diagnosed with NSCLC, the prognosis is relatively poor, with 5-year survival rates varying depending on the stage of the disease—from 68% at early stages (IB stage) to 10% at late stages (IVA, IVB) [3]. Therefore, novel therapies to improve prognosis are warranted. The identification of driver gene mutations has paved the way for targeted treatments, significantly enhancing survival outcomes in patients. Currently, key druggable targets include epidermal growth factor receptor (EGFR), anaplastic lymphoma kinase (ALK), v-raf murine sarcoma viral oncogene homolog B (BRAF), ROS proto-oncogene 1 (ROS1), Kirsten rat sarcoma virus (KRAS), human epidermal growth factor receptor 2 (HER2), c-MET proto-oncogene (MET), neurotrophic receptor tyrosine kinase (NTRK), rearranged during transfection (RET), and neuregulin 1 (NRG1). Additionally, phosphoinositide 3-kinases (PI3K) and fibroblast growth factor receptor (FGFR) are emerging targets being actively explored in clinical trials [4, 5].

TNF-related apoptosis-inducing ligand (TRAIL) receptors provide interesting targets for therapy. TRAIL is an apoptosis-inducing ligand belonging to the TNF superfamily. In contrast to most other TNF family members, TRAIL is expressed in a broad range of tissues and is presumably an important role in the immune response against tumor cells [6]. In addition, TRAIL-induced apoptosis is highly selective for tumor cells and usually non-toxic to normal cells [7]. The binding of TRAIL to its death receptors, DR4 (TRAIL-R1) and DR5 (TRAIL-R2), results in trimerization of the receptors and subsequently the activation of both the extrinsic and the intrinsic apoptotic pathways. After trimerization of the receptors, the death-inducing signaling complex (DISC) is formed, containing FADD and procaspase-8, resulting in caspase-8 cleavage and activation. Cellular FLICE-like inhibitory protein (cFLIP) is structurally related to caspase-8 and can bind to the DISC, blocking the association of caspase-8 with the DISC and preventing subsequent activation [8]. Activated caspase-8 can either directly activate effector caspase-3 via the extrinsic apoptotic pathway (type I cells) or can cross-talk with the intrinsic apoptotic pathway via the cleavage of Bid into truncated Bid (tBid) (type II cells) [9]. This will lead to the oligomerization of pro-apoptotic proteins Bak and Bax, which disrupt the mitochondrial membrane potential, resulting in the release of cytochrome c and Smac/DIABLO [10]. Smac/DIABLO neutralizes the function of X-linked inhibitor of apoptosis protein (XIAP), which normally exerts its anti-apoptotic function via binding to activated caspase-9 and -3 [11]. Cytochrome c then forms together with APAF-1 and pro-caspase-9 the apoptosome, leading to the activation of caspase-9, which in turn results in caspase-3 activation and subsequent apoptosis induction [12].

Recombinant human (rh)TRAIL has been tested in clinical NSCLC studies as a single anti-tumor agent, with the best response being stable disease [13]. Based on potentiating effects in preclinical studies, TRAIL-receptor targeting therapies were also combined with other therapeutic drugs [12, 14]. One of those combinational drugs is bortezomib, which is a reversible proteasome inhibitor approved for the treatment of multiple myeloma and mantle cell lymphoma. In a preclinical setting, bortezomib has been demonstrated to increase TRAIL-receptor levels and enhance rhTRAIL-mediated apoptosis induction [15–17]. However, rhTRAIL-induced apoptosis is mechanistically different between type I and type II cells, and the mechanisms of sensitization by bortezomib on either extrinsic or intrinsic apoptosis signaling need further clarification.

Recent studies have highlighted the relevance of regulated cell death pathways other than apoptosis, most notably necroptosis, in shaping the cellular response to TRAIL. Necroptosis is a caspase-independent form of programmed cell death mediated by the activation of RIPK1, RIPK3, and MLKL, ultimately leading to plasma membrane permeabilization and a pro-inflammatory cell death phenotype [18]. When caspase-8 is missing or its function is blocked, TRAIL can induce necroptosis rather than apoptosis by forming a signaling complex composed of RIPK1, RIPK3, and MLKL. In NSCLC and other cancers, the loss of caspase-8 activity or disturbances in necroptosis-related proteins have been associated with resistance to treatment and can affect how cells respond to TRAIL therapy [7]. Consequently, necroptosis represents a mechanistically and clinically relevant alternative or complementary pathway to apoptosis, yet its interaction with bortezomib/TRAIL co-treatment remains unexplored.

In our previous study, we explored the apoptotic mechanisms behind TRAIL sensitivity in bortezomib-resistant NSCLC, including A549 cells. Interestingly, we found that generated bortezomib-resistant A549 cells switched to rhTRAIL sensitive phenotype [19]. We also explored extensively the time-course of changes in signaling pathways upon TRAIL exposure [20–22]. In the current study, the mechanisms underlying bortezomib-mediated sensitization to TRAIL via the intrinsic and extrinsic apoptosis pathways were further explored in NSCLC cells. We focused primarily on signaling pathways shown to be important in TRAIL downstream effects [20, 21] and the roles of cFLIP, Bid, and XIAP, along with monitoring the impact of activation of different caspases in rhTRAIL-induced apoptosis.

## Materials and methods

### Cell lines

The NSCLC cell lines H460, A549, and SW1573 were obtained from the American Type Culture Collection (ATCC) (Manassas, VA). The cell lines were tested annually for authenticity by short tandem repeat profiling DNA fingerprinting (Baseclear, Leiden, The Netherlands) and for mycoplasma by PCR. Cells were only used when mycoplasma negative. H460 and A549 cells were cultured in RPMI 1640 medium (Gibco, Paisley, Scotland), and SW1573 cells in DMEM medium (Gibco). Culture media were supplemented with 10% fetal calf serum (FCS) (Sanbio, Uden, The Netherlands), 100 U/mL penicillin, and 100 µg/mL streptomycin (Invitrogen, Breda, The Netherlands). Cells were grown at 37°C in a humidified atmosphere containing 5% CO_2_. Cell lines were harvested by trypsinization and passaged twice weekly.

### Reagents

RhTRAIL, TRAIL-R1 specific variant (S159R) [23], and TRAIL-R2 specific variant (D269H/E195R) [24] were produced non-commercially as described earlier [25]. Bortezomib (Velcade™, Janssen-Cilag BV, Tilburg, The Netherlands) was provided as a pure substance and dissolved in 0.9% sodium chloride. Primary antibodies for cleaved caspase-8, -9, and -3, and Bid were purchased from Cell Signaling Technology (Danvers, MA, USA). Primary antibody for cFLIP was from Transduction Laboratories (Lexington, KY, USA), and mouse anti-XIAP clone 2F1 from MBL International (Woburn, USA). Mouse anti-β-actin was purchased from Sigma-Aldrich (Zwijndrecht, The Netherlands). The secondary antibodies used were goat-a-mouse-IRDye (1:10,000, 800CW; #926-32210 and 680; #926-32220), or goat-a-rabbit-IRDye (1:10,000, 800CW; #926-32211 and 680; #926-32221), all purchased from LI-COR Biosciences (Lincoln, Nebraska, USA).

### Western blot analysis

Western blot analysis was performed as previously described [21, 22, 26]. In brief, the cells were disrupted in lysis buffer (Cell Signaling Technology Inc., Danvers/Boston, MA, USA), protein was separated on an SDS-PAGE, and electroblotted onto polyvinylidene difluoride (PVDF) membranes (Millipore, Amsterdam, The Netherlands). Next, membranes were blocked for one hour at room temperature in InfraRedDye blocking buffer (Rockland Inc., Pennsylvania, USA) and incubated overnight at 4°C with the primary antibodies diluted in 1:1 InfraRedDye blocking buffer with PBS-T (PBS with 0.05% Tween-20). The membrane was incubated with the secondary antibodies for one hour at room temperature in the dark. Protein expression was detected using the Odyssey Infrared Imager (LI-COR Biosciences, Lincoln, Nebraska, USA), 84 µm resolution, 0 mm offset, and high quality.

### Detection of apoptosis by FACS analysis

Apoptosis was measured using the FITC Annexin V Apoptosis Detection kit 1 (BD Biosciences, Franklin Lake, USA) according to the manufacturer’s protocol. Briefly, after exposure to rhTRAIL and/or bortezomib, cells were harvested and washed with cold PBS. Following resuspension in 1× binding buffer, 5 µL of FITC Annexin V and 10 µL of propidium iodide solution were added. Cells were incubated for 15 minutes at room temperature in the dark. After the addition of 400 µL 1× binding buffer, apoptosis was analyzed by flow cytometry using the FACS Calibur (Becton Dickinson) with an acquisition of 10,000 events. Cells were considered non-apoptotic (Annexin V-FITC negative/PI negative), in early apoptotic phase (Annexin V-FITC positive/PI negative), in late apoptotic phase (Annexin V-FITC positive/PI positive), or dead (Annexin V-FITC negative/PI positive).

### Clonogenic survival

Clonogenic survival was performed as described previously [26]. Concentrations of the drugs were based on drug sensitivity experiments (performed by using the MTT assay and clonogenic survival) as described earlier [19, 26]. Briefly, cells were seeded at a density of 250 cells/well and treated with 100 ng/mL TRAIL and/or 50 nM Bortezomib. After 6 hours of exposure, the medium was refreshed, and the cells were incubated for 1–2 weeks to form colonies. Upon the formation of colonies, cells were washed with PBS, fixed with 99% ethanol, and washed again. Colonies were stained with 10% Giemsa (Merck, Darmstadt, Germany), and colonies consisting of > 50 cells were counted. The surviving fraction was determined by dividing the number of colonies by the number of cells plated. The surviving fraction of untreated cells was set to 1.

### Flow cytometric analysis of cell cycle distribution

Cell cycle analysis and cell death measurements were performed as described previously [27]. Briefly, 100,000 cells were seeded in a 6-well plate. After exposure to TRAIL, bortezomib, or their combination, cells were trypsinized, resuspended in medium collected from the matching sample, and centrifuged for 5 min at 1,200 rpm. Cells were washed with PBS and subsequently stained with propidium iodide-containing buffer [50 µg/mL propidium iodide, 0.1% (Tri-) Sodium Citrate, 0.1% Triton X-100, 0.1 mg/mL RNAse]. DNA content was analyzed by a FACS Calibur (Becton Dickinson) with an acquisition of 10,000 events. Cell death was quantified by calculating the area of the sub-G1 peak.

### RNA interference

Sequences for small interfering RNA (siRNA) molecules were for Bid: 5’-GAA UAG AGG CAG AUU CUG AdTdT-3’ (sense) and 5’-UCA GAA UCU GCC UCU AUU CdTdT-3’ (anti-sense), for XIAP: 5’-GUG GUA GUC CUG UUU CAG CdTdT-3’ (sense) and 5’-GCU GAA ACA GGA CUA CCA CdTdT-3’ (anti-sense) and for cFLIP: 5’-GAG GUA AGC UGU CUG UCG GdTdT-3’(sense) and 5’-CCG ACA GAC AGC UUA CCU CdTdT-3’(anti-sense) as used before [28]. The siRNA control, without any known homology with the human genome, was purchased from Eurogentech (Seraing, Belgium). H460, A549, and SW1573 cells were transfected in 6-well plates with 10 µL of 20 µM siRNA duplexes using Oligofectamine reagent according to the manufacturer’s instructions (Invitrogen BV, Breda, The Netherlands). After 24 hours, cells were replated for apoptosis assay (96-well culture plate).

### Apoptosis assay

For apoptosis measurements, 3,000 cells were plated in 96-well culture plates. Cells were continuously incubated with bortezomib and/or rhTRAIL or TRAIL-receptor specific variants (S159R and D269H/E195R) at various concentrations, which were chosen based on data published earlier [19, 26]. Staining was performed 6 or 24 hours after the start of incubation. Acridine orange (5 mg/mL) was added to each well to distinguish apoptotic cells from viable cells. Staining pattern was determined by fluorescence microscopy, where apoptosis was defined as the appearance of apoptotic bodies and/or chromatin condensation. Results are expressed as the percentage of apoptotic cells in a culture by counting at least 300 cells per well.

### Statistics

All experiments were performed at least in triplicate. Data were analyzed by the student’s *t*-test for unpaired data; in cases appropriate, we used a paired *t*-test; *p*-values < 0.05 were considered statistically significant, and exact numbers are provided in the legends. In case no specification is given, there is no statistical difference. All data were normally distributed, and only comparisons between two groups were evaluated statistically. Statistical analysis was performed using GraphPad Prism (version 5.0 GraphPad Software, San Diego, CA, USA).

## Results

### Rapid caspase activation in NSCLC cells after rhTRAIL treatment

**Figure 1:**
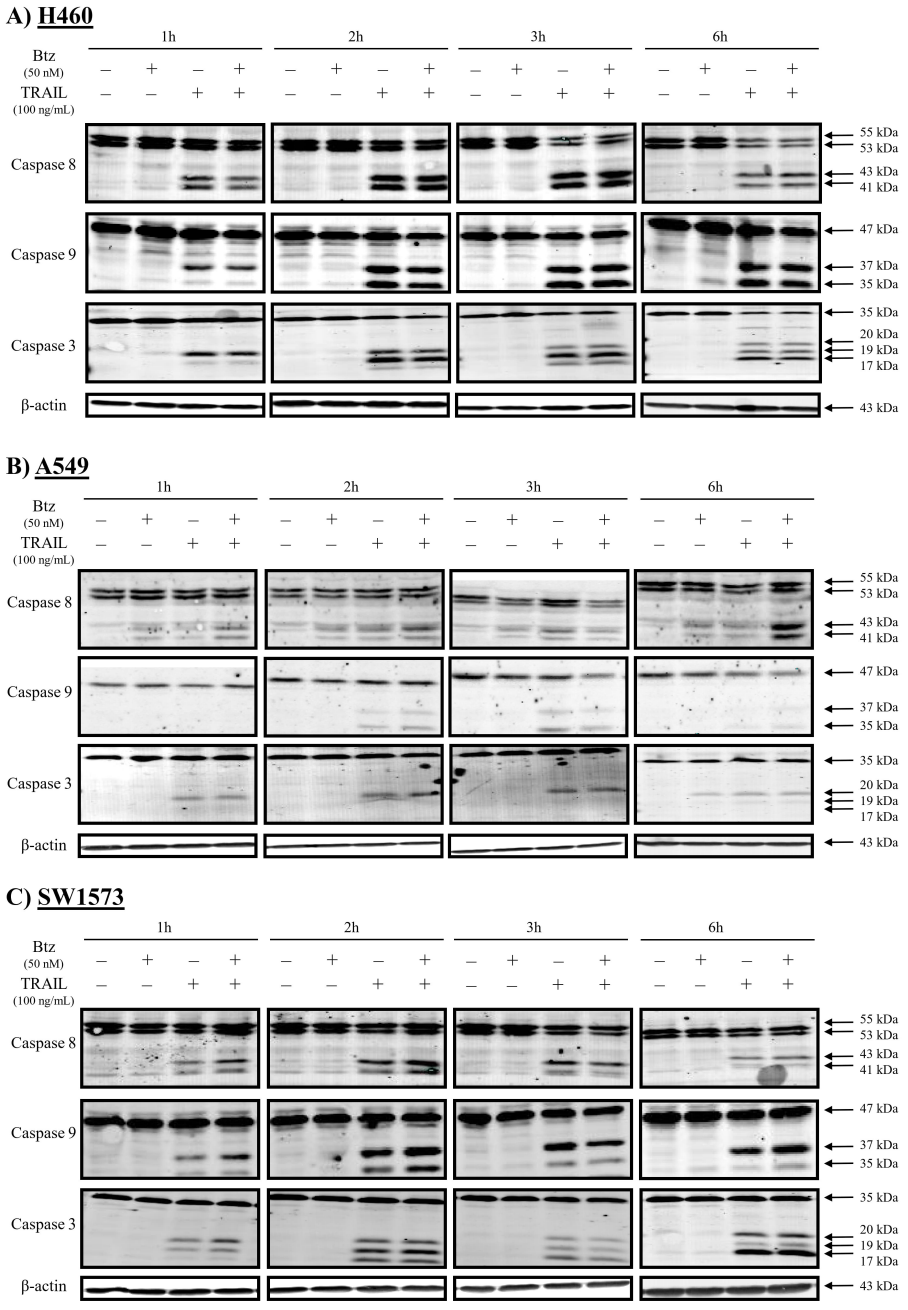
**Rapid caspase activation in NSCLC cells after treatment with rhTRAIL.** Western blot analysis of caspases-8, -9 and -3 in H460 (**A**), A549 (**B**) and SW1573 cells (**C**) after 1-, 2-, 3- and 6-hours treatment with 50 nM bortezomib and/or 100 ng/mL rhTRAIL. A representative blot of at least 3 different experiments is shown. β-actin served as a loading control.

Since TRAIL rapidly induces apoptosis, we determined caspase activation at early time points (1, 2, 3, and 6 hours) in TRAIL-sensitive H460 and TRAIL-resistant A549 and SW1573 NSCLC cells upon exposure to bortezomib and/or rhTRAIL (Figure 1). Bortezomib as a single agent did not induce caspase cleavage within 6 hours of treatment in the 3 cell lines tested. In sensitive H460 cells, the combination of bortezomib with rhTRAIL did not affect the kinetics of rhTRAIL-induced caspase-8, -9, and -3 activation (Figure 1A). Caspase cleavage in these cells was already detectable after 1 hour, and increased at later time points upon rhTRAIL and combined treatment. A p37 cleavage product of caspase-9 was detected after 1 hour treatment, followed by a strong increase in both p37/35 cleavage products at later time points. Since caspase-9 p37 is generated by caspase-3-dependent cleavage, this suggests early initiation of the extrinsic apoptotic pathway and subsequent caspase-9 cleavage [29]. The p35 product, detected after 2 hours of treatment, has been linked with auto-cleavage of caspase-9 via the intrinsic pathway. Caspase-3 cleavage was initially marked by the appearance of p20 and p19 cleavage products at 1 hour post-treatment. By 6 hours, a clear increase in the p17 cleavage product, representing a more active form of caspase-3, was observed along with a concomitant decrease in full-length caspase-3 (Figure 1A).

In contrast to H460 cells, A549 cells displayed overall lower levels of caspase cleavage upon bortezomib and/or rhTRAIL treatment. Particularly, combined treatment induced weak caspase-8 cleavage that increased further at 6 hours post-treatment (Figure 1B). Only weak caspase-9 cleavage was detected following treatment with rhTRAIL alone or combined with bortezomib. Also, marginal caspase-3 cleavage was observed, although this did not yield the p17 product (Figure 1B). Cleavage of these caspases was detected up to 3 hours post-treatment and was hardly detectable after 6 hours.

Finally, in SW1573 cells (Figure 1C), exposure to rhTRAIL caused rapid cleavage of caspase-8, although at lower levels than seen in H460 cells. Caspase-8 cleavage was slightly increased upon combined rhTRAIL/bortezomib treatment. Furthermore, caspase-9 cleavage yielded mainly the p37 product that is generated by caspase-3-mediated caspase-9 cleavage. The p17 cleaved fragment of caspase-3 was detectable at 6 hours post-treatment, although a large proportion of full-length caspase-3 remained uncleaved (Figure 1C). In summary, rhTRAIL-induced caspase-8 cleavage is detectable at early time points in all cell lines, with high levels observed in sensitive cells and low levels in resistant cells. Combination treatment with bortezomib slightly increased caspase-8 cleavage. The cleavage patterns of caspase-3 and -9 were markedly different between the sensitive and resistant NSCLC cell lines, as well as between the resistant cells. Since in another study, the pan-caspase inhibiter zVAD completely prevented induction of cell death [30] and the caspase-8 inhibitor zIETD partially (manuscript submitted), we concluded that in these cell lines cell death was caspase dependent and we did not further investigate non-caspase induced cell death.

### Combined rhTRAIL/bortezomib treatment triggers apoptotic cell death and inhibits clonogenic growth of NSCLC cells

**Figure 2:**
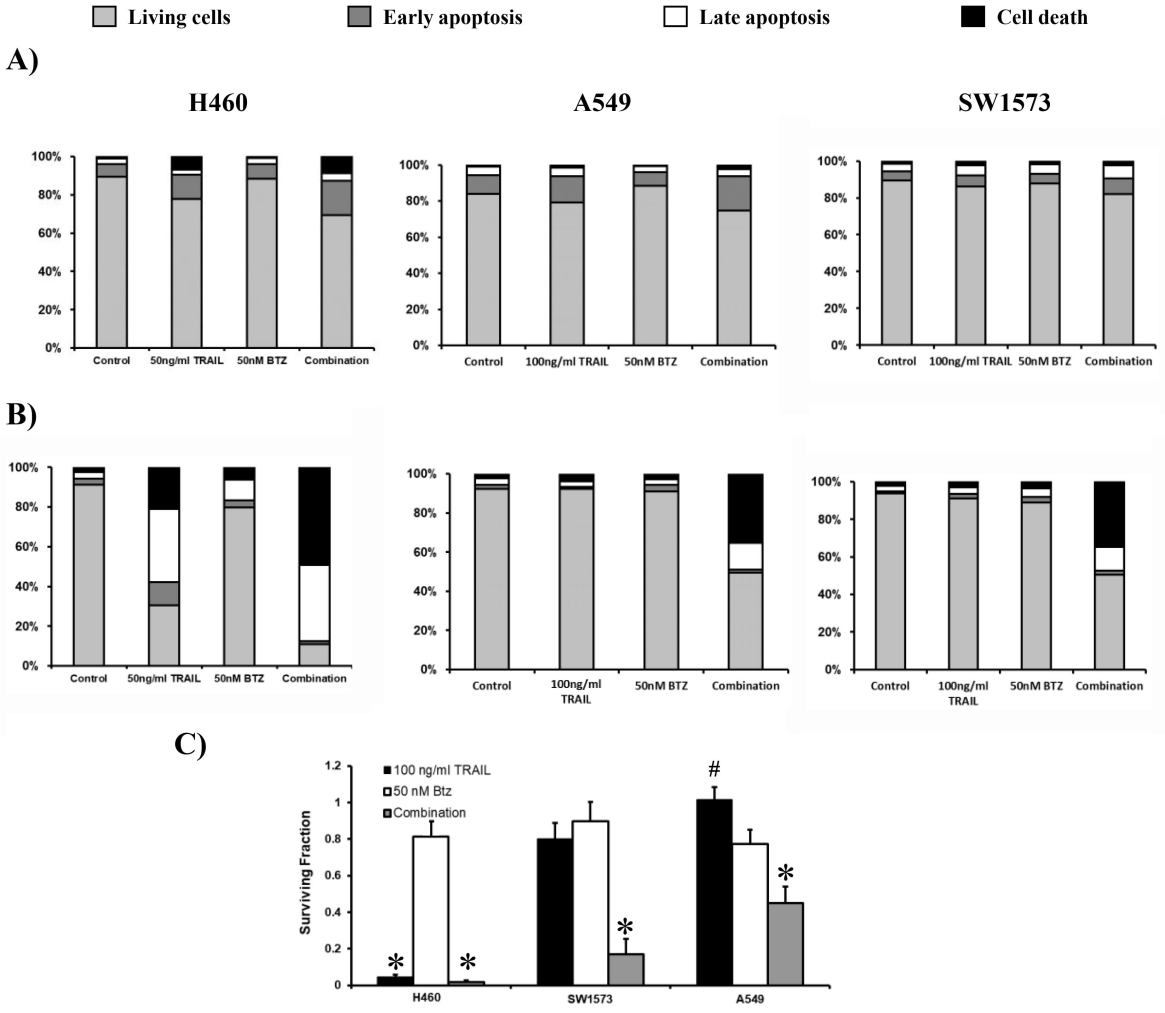
**Bortezomib (BTZ) potentiates rhTRAIL-induced apoptosis in sensitive and resistant NSCLC cells.** (**A**) Cell lines H460, A549 and SW1573 were treated with rhTRAIL (50 or 100 ng/mL for H460 and A549/SW1573, respectively) and/or 50 nM BTZ for 6 hours. After staining with Annexin V and PI, cells were evaluated for apoptosis. (**B**) H460, A549 and SW1573 cells were treated for 6 hours (similar to **A**), followed by incubation in drug-free medium for an additional 18 hours, after which apoptosis was measured. (**C**) Clonogenic outgrowth was determined after treatment for 6 hours with 100 ng/mL rhTRAIL and/or 50 nM BTZ, followed by incubation in drug-free medium for 1–2 weeks. Colony growth is depicted relative to the untreated control cells (surviving fraction 1). Values are means ± SD of at least three independent experiments. By default, comparison to untreated controls, *: *p* < 0.0001 (and in H460 cells to BTZ alone; in A549, the combination versus TRAIL or BTZ alone), #: *p* < 0.001 (in SW1573, comparison between the combination and TRAIL alone). Data in **A** and **B** are means of at least 3 separate experiments with an SD being less than 10%.

To determine whether caspase activation leads to apoptosis induction in H460, A549, and SW1573 cells, FACS analysis was performed using Annexin V and propidium iodide (PI). This approach enabled the identification of early apoptotic cells (Annexin V-positive/PI-negative), late apoptotic cells (Annexin V-positive/PI-positive), and necrotic or late-stage apoptotic cells (PI-positive only). Apoptosis was first examined after 6 hours of treatment. As expected, H460 cells were sensitive to 50 ng/mL rhTRAIL, showing early (13%) and late (3%) apoptosis and dead cells (7%) (Figure 2A). Bortezomib at 50 nM did not trigger cell death, whereas combined rhTRAIL/bortezomib exposure resulted in 18% early, 4% late apoptosis, and 9% dead cells. In A549 cells, 100 ng/mL rhTRAIL and/or bortezomib exposure resulted in similar levels of early and late apoptosis as seen in H460 cells, however, no cell death was induced within 6 hours of exposure (Figure 2A). SW1573 cells showed no induction of apoptosis and cell death after rhTRAIL or combined treatment (Figure 2A).

Next, we assessed apoptosis by maintaining the cells for 18 hours following the initial 6-hour exposure to bortezomib and/or rhTRAIL. The exposure of H460 cells to rhTRAIL alone resulted in 12%, 37% and 21% of early-, late-apoptotic and dead cells, respectively. The combination of bortezomib and rhTRAIL strongly increased late apoptotic (38%) and dead cells (49%). Both A549 and SW1573 cells displayed minimal induction of apoptosis when exposed to either rhTRAIL or bortezomib alone. However, combined rhTRAIL/bortezomib treatment showed around 15% late apoptotic cells and 35% dead cells (Figure 2B). The antitumor effect of rhTRAIL, bortezomib and combined treatment was also tested in clonogenic assays. In all three NSCLC cells, bortezomib had little effect (Figure 2C). Clonogenic growth was almost completely suppressed by rhTRAIL and combined treatment in H460 cells, whereas in A549 and SW1573 cells, only combined treatment significantly reduced clonogenic growth. Thus, 6 hours combined rhTRAIL/bortezomib treatment is sufficient to initiate apoptotic cell death at later time points and was able to strongly suppress clonogenic growth.

### Bortezomib differentially sensitizes cells to rhTRAIL in a cell specific manner

**Figure 3:**
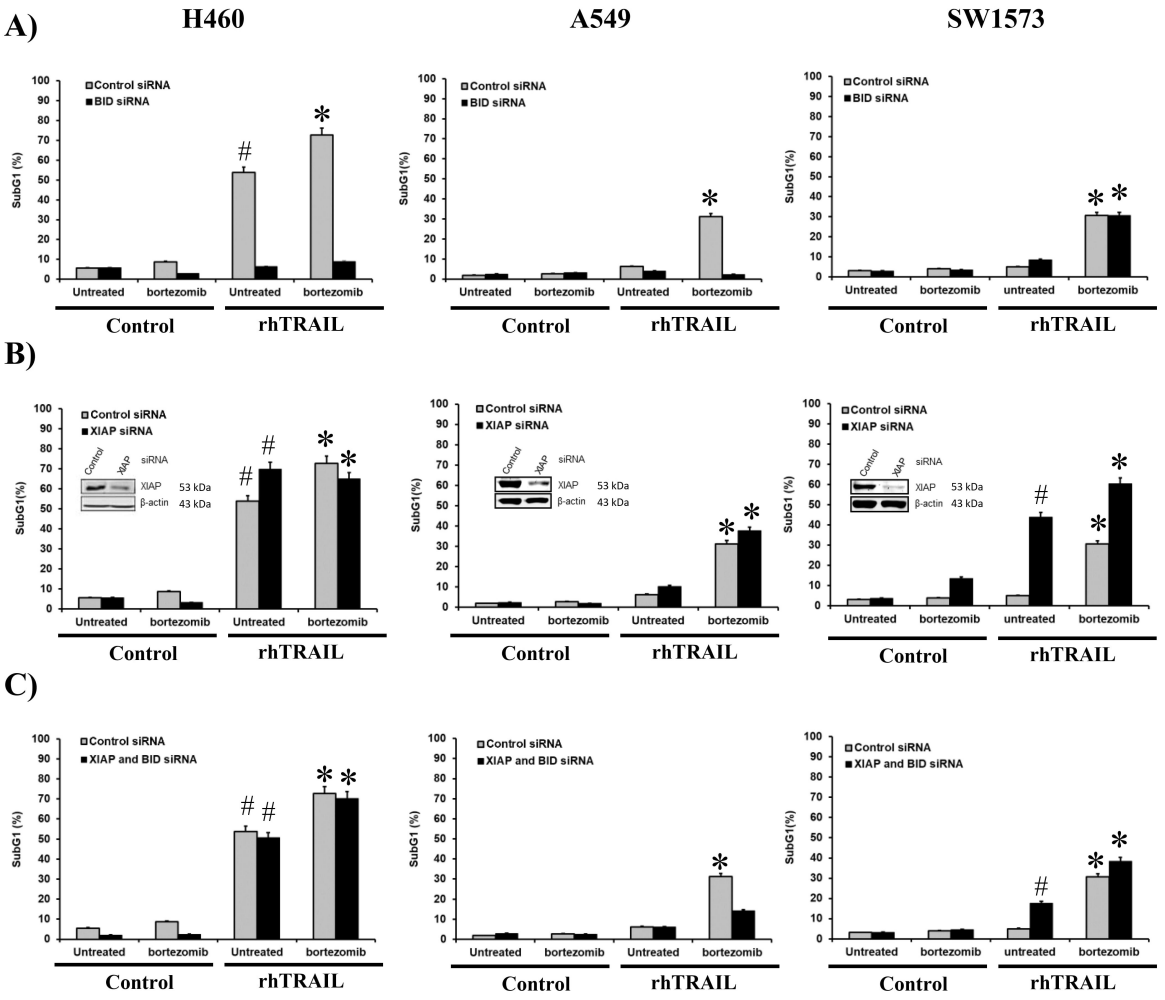
**The bortezomib (BTZ) sensitizing effect in H460 and A549 cells is predominantly mediated via the caspase-8/Bid amplification loop whereas mainly the extrinsic pathway is enhanced in SW1573 cells.** H460, A549 and SW1573 cells were transfected with Bid siRNA (**A**), XIAP siRNA (**B**) and both Bid and XIAP siRNA (**C**). At 48 hours after transfection, cells were exposed to 50 ng/mL rhTRAIL and/or 25 nM BTZ for 24 hours, followed by cell death measurement. #: *p* < 0.01 [rhTRAIL vs. control (–BTZ)]. *: *p* < 0.01 [combination treatment with rhTRAIL + BTZ vs. control (+BTZ)].

To investigate the mechanisms underlying bortezomib-dependent sensitization to TRAIL, we first explored the involvement of the extrinsic and intrinsic apoptosis pathways by inhibiting Bid. The cleavage and activation of Bid by caspase-8 connect the extrinsic- with the intrinsic apoptotic pathway, the so-called amplification loop. The three NSCLC cell lines were treated continuously with rhTRAIL, bortezomib or the combination for 24 hours. rhTRAIL resulted in over 50% cell death (measured as the sub-G1 fraction) in H460, while A549 and SW1573 showed less than 10% apoptosis (Figure 3A). Combined rhTRAIL/bortezomib treatment strongly increased cell death to approximately 30% in A549 and SW1573 cells and 70% H460 cells. Silencing of Bid strongly reduced rhTRAIL-induced cell death in H460 cells from around 55% to 5%, whereas no significant effects were seen in A549 and SW1573 cells. Upon combined treatment Bid silencing strongly inhibited cell death in H460 cells (Figure 3A). In A549 cells cell death activation by combined rhTRAIL/bortezomib exposure was effectively inhibited in Bid silenced cells. Interestingly, in SW1573 cells Bid silencing had no effect on rhTRAIL/bortezomib induced cell death. Together, these findings indicate that bortezomib sensitizes rhTRAIL induced apoptosis predominantly via the Bid-dependent amplification loop in H460 and A549 cells, whereas in SW1573 cells sensitization occurs by stimulating the extrinsic apoptotic pathway.

Since we observed distinct cleavage patterns for caspase-9 and caspase-3 in the 3 cell lines (Figure 1). We also examined the involvement of the caspase-9 and -3/-7 inhibitor XIAP in bortezomib-dependent sensitization. Silencing of XIAP increased rhTRAIL-induced cell death further in H460 cells from around 55% to 70% (Figure 3B). Interestingly, the somewhat increased level of cell death after combined treatment did not increase further after XIAP silencing, and showed actually some decrease in apoptosis. In XIAP-silenced A549 cells, no significant increase in rhTRAIL but a significant increase in rhTRAIL/bortezomib-induced cell death was seen compared to control siRNA transfected cells. Interestingly, in SW1573 cells XIAP silencing strongly enhanced rhTRAIL-induced cell death from around 5% to 45%. Also combined treatment increased cell death from around 30% to 60%. This suggests that in SW1573 cells, the intrinsic pathway is suppressed by XIAP, and the bortezomib-induced enhancement, primarily mediated by the extrinsic pathway, is further amplified by the derepression of the intrinsic apoptosis pathway.

Next, we examined the combined involvement of Bid and XIAP in bortezomib-dependent rhTRAIL sensitization, hence, under conditions in which the caspase-8/Bid amplification loop is inhibited and caspase-9 and downstream caspases are derepressed. Whereas Bid silencing suppressed rhTRAIL and rhTRAIL/bortezomib induced apoptosis in H460 cells, combined Bid/XIAP depletion restored cell death levels to around 50% and 70%, respectively (Figure 3A, C). Depression of the caspases appears sufficient to trigger effective rhTRAIL-induced apoptosis, even when the Bid-dependent pathway is disrupted. In A549 cells, where bortezomib sensitization was also dependent on the Bid-amplification loop, simultaneous silencing Bid and XIAP resulted in higher levels of apoptosis than seen upon Bid depletion alone. This resembles that of H460 cells, although apoptotic levels are obviously lower in these resistant cells. Silencing of Bid/XIAP in SW1573 cells resulted in similar apoptosis levels as seen in Bid-depleted cells and around 20% lower than in XIAP silenced cells (Figure 3A, B, C). This suggests that although rhTRAIL/bortezomib induced apoptosis in SW1573 is mainly dependent on the extrinsic apoptotic pathway, derepression of caspase-9 and downstream caspases will further increase apoptosis.

### Bortezomib sensitizes rhTRAIL-induced apoptosis downstream of caspase-8 activation

**Figure 4:**
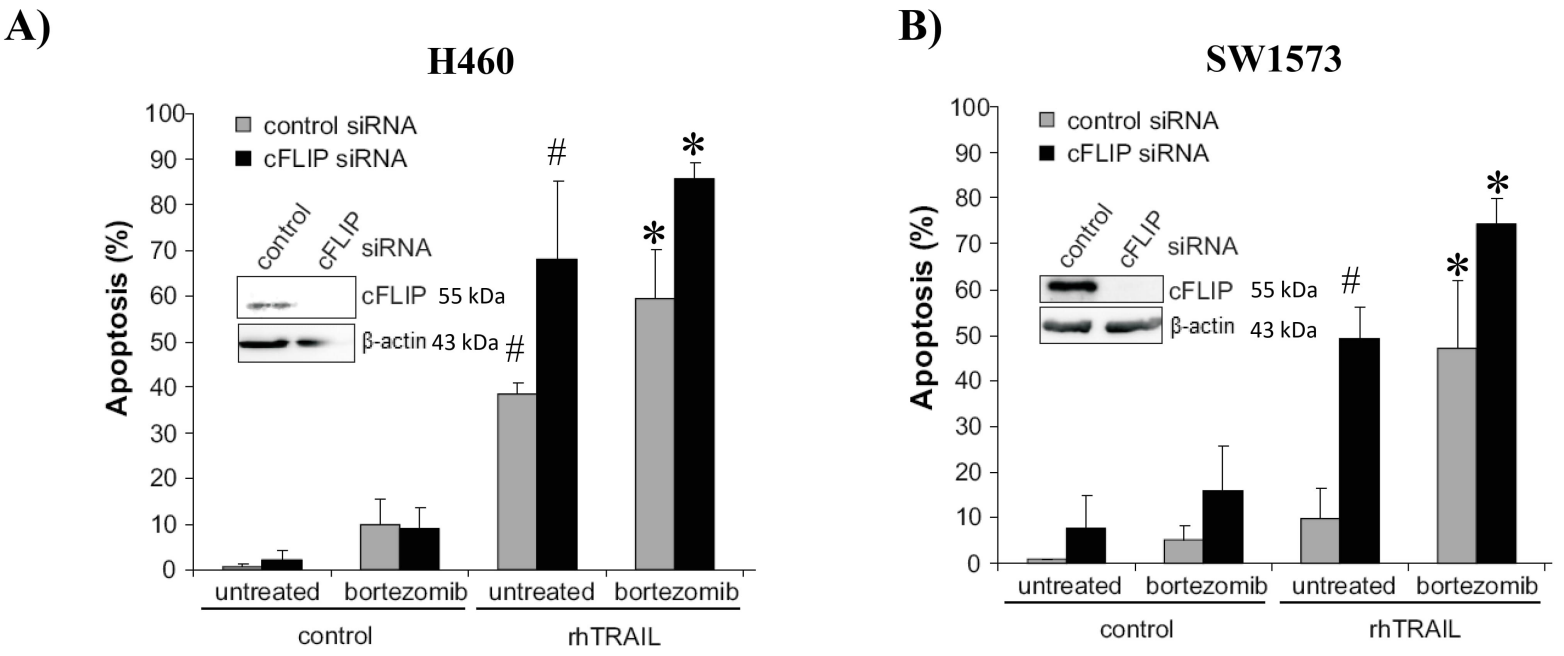
**cFLIP suppression sensitizes rhTRAIL induced apoptosis with and without bortezomib (BTZ).** H460 (**A**) and SW1573 (**B**) cells were transfected with cFLIP siRNA. After 48 hours, cells were treated with BTZ (50 nM) and/or rhTRAIL (50 ng/mL) for 24 hours, after which apoptosis was evaluated. *: *p* < 0.01 (combination treatment with rhTRAIL + BTZ vs. control); #: *p* < 0.01 (rhTRAIL vs control).

We next investigated the possible involvement of cFLIP in regulating rhTRAIL-induced apoptosis and sensitization to bortezomib. cFLIP is a potent inhibitor of extrinsic apoptosis by blocking caspase-8 activation. In H460 cells, silencing of cFLIP markedly increased rhTRAIL-induced apoptosis from around 40% to 70%. Also, upon combined rhTRAIL/bortezomib treatment, apoptosis levels increased from 60% to 85% in cFLIP siRNA-transfected cells (Figure 4A). In SW1573 cells, down-regulation of cFLIP strongly increased rhTRAIL-induced apoptosis from 10% to 50%, indicating that cFLIP is a major determinant of rhTRAIL-resistance in these cells (Figure 4B). Combined rhTRAIL/bortezomib exposure resulted in an additional increase in apoptosis levels from 50% to 75% in cFLIP-suppressed SW1573. Taken together, this indicates that in both NSCLC cells, caspase-8 activation is an important rate limiting step for rhTRAIL-induced apoptosis, which can be overcome by bortezomib. Bortezomib also enhanced apoptosis in cFLIP silenced cells, indicating sensitizing effects also downstream of caspase-8 activation.

## Discussion

**Figure 5:**
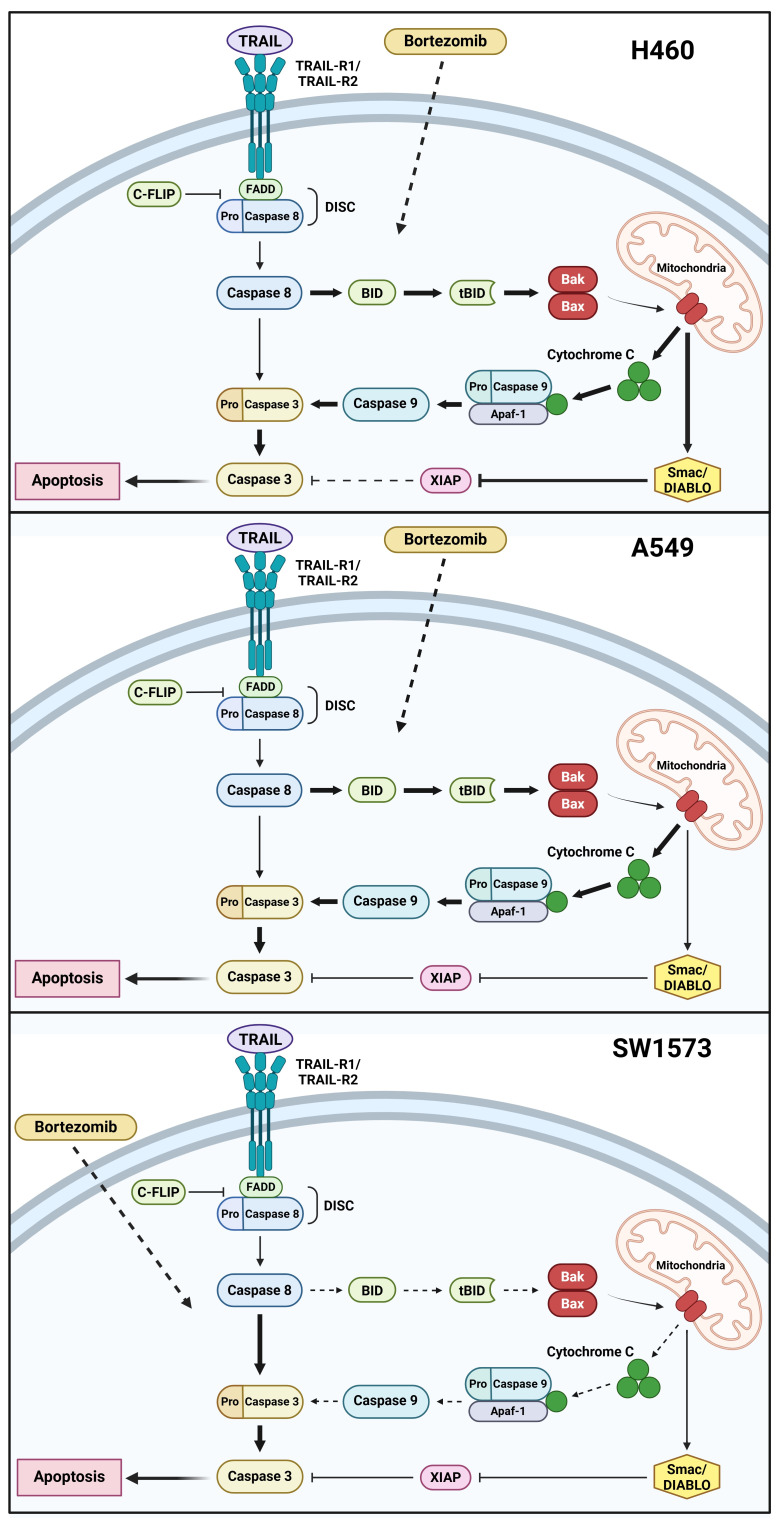
**Schematic representation of the bortezomib sensitizing effect on rhTRAIL induced apoptosis in NSCLC cells.** Bortezomib enhances rhTRAIL-induced apoptosis by modulating either the extrinsic or intrinsic apoptotic pathways (indicated by the dashed arrows for bortezomib). Inhibition is indicated by blunt-ended arrows. rhTRAIL-induced apoptosis involves both intrinsic and extrinsic pathways in H460 cells, with bortezomib enhancing apoptosis through the caspase-8/Bid signaling loop (indicated by fat arrows and decreased XIAP inhibition indicated by dashed blunt-added arrow). In A549 cells, TRAIL alone triggers a weak extrinsic apoptotic response (thin arrows), whereas bortezomib significantly amplifies this effect via the same caspase-8/Bid pathway (fat arrows). In SW1573 cells, bortezomib primarily facilitates rhTRAIL-induced apoptosis through the extrinsic apoptotic pathway (dashed arrows for the intrinsic pathway; fat arrows for the extrinsic pathway). Created in BioRender. Anielska, A. (2025) https://BioRender.com/6v0bvz3. Paweł Kochany has obtained permission to reuse the figure.

In this study, we aimed to determine the molecular mechanisms through which rhTRAIL elicits a differential apoptotic response in NSCLC cells and how bortezomib may potentiate rhTRAIL efficacy. Interestingly, the molecular mechanisms underlying bortezomib sensitization to rhTRAIL seemed to differ among individual NSCLC cell lines. A schematic representation of our findings is depicted in Figure 5.

In H460 cells, the inhibition of Bid suppressed apoptosis induction, indicating that apoptosis triggered by rhTRAIL and bortezomib treatment primarily relied on the mitochondrial apoptotic pathway. However, Bid and XIAP suppression restored the level of apoptosis, suggesting that not the cleavage of caspase-9 but the release of Smac/DIABLO via the mitochondria was crucial, indicating a blockage of the extrinsic apoptotic pathway. This was in line with previous findings underlining the pivotal role of the mitochondria by Bcl-2 overexpression and Smac/DIABLO release [31]. Caspase cleavage patterns show caspase-9 cleavage by caspase-3, followed by auto-cleavage of caspase-9, which also implies the importance of the extrinsic apoptotic pathway. Moreover, since bortezomib does not enhance rhTRAIL-mediated apoptosis induction upon XIAP suppression, bortezomib most likely exerts its rhTRAIL sensitizing effect via the caspase-8 activation and the Bid-dependent amplification loop.

In rhTRAIL-resistant A549 cells, suppression of Bid sufficiently inhibited bortezomib-mediated rhTRAIL-induced apoptosis. Additionally, reduction of XIAP did not affect rhTRAIL-induced apoptosis in the absence or presence of bortezomib, indicating that rhTRAIL-induced apoptosis was not affected by XIAP. This is in contrast with a previous study reporting the importance of enhanced cleavage of XIAP for increased rhTRAIL-induced apoptosis mediated by proteasome inhibitor MG132 in cervical cancer cells [32]. Moreover, following treatment with a combination of bortezomib and rhTRAIL, downregulation of XIAP expression was observed in rhTRAIL-resistant colon cancer cells [33]. Knock-down of both Bid and XIAP demonstrated caspase-9 activation to be crucial for rhTRAIL-induced apoptosis, instead of the release of Smac/DIABLO as was seen in H460 cells. These findings are consistent with the western blot analysis, which reveals the presence of the p35 cleavage product of caspase-9.

The extrinsic apoptotic pathway appears to be crucial for the induction of apoptosis by rhTRAIL and bortezomib in rhTRAIL-resistant SW1573 cells. In these cells, suppression of Bid had no effect. However, in line with a previous study [32], XIAP appeared to be a main determinant for rhTRAIL sensitivity in SW1573 cells. The role of the extrinsic apoptotic pathway was also confirmed by the p37 cleavage product of caspase-9 upon bortezomib and rhTRAIL exposure. However, the reduction of both XIAP and Bid demonstrated that some caspase-9 cleavage is required for enhanced apoptosis.

Suppression of cFLIP demonstrated that caspase-8 activation is an important rate limiting step for rhTRAIL-induced apoptosis in H460 and SW1573 cells. Enhanced levels of cleaved caspase-8 as a mechanism of rhTRAIL sensitization by bortezomib has been described previously [34, 35]. Bortezomib exposure, however, was shown to enhance cFLIP levels in several cell lines including, H460 and other NSCLC cells [15, 36]. This increase of cFLIP expression after bortezomib treatment implies that the increase of TRAIL-receptor levels and improved DISC formation should be even more pronounced to outbalance the enhanced cFLIP levels. In hepatoma cells, primary glioma and esophageal squamous cell carcinoma cells DISC analysis revealed that bortezomib enhanced the recruitment of TRAIL-receptors, FADD and caspase-8 upon rhTRAIL stimulation [35, 37, 38]. Although bortezomib exposure reduced cFLIP levels in these cell lines, enhanced recruitment of cFLIP to the DISC was still observed. Nevertheless, this enhancement was outweighed by the enhanced recruitment of TRAIL-receptors and caspase-8 to the DISC, therefore the net effect was an increased caspase-8/cFLIP ratio at the DISC [37, 38]. These results demonstrate that cFLIP has a high binding affinity for the DISC allowing efficient inhibition of apoptosis even when present at low concentrations as they were found in SW1573 cells. However, cFLIP downregulation combined with bortezomib further sensitized H460 and SW1573 cells to rhTRAIL. This indicates that bortezomib also exerts its effect downstream of caspase-8 activation.

In summary, we found that bortezomib-induced sensitization to rhTRAIL primarily proceeds via either the extrinsic or intrinsic apoptosis pathway as controlled by Bid, XIAP and cFLIP. However, bortezomib affects multiple proteins involved in rhTRAIL-induced apoptosis in NSCLC cells as well as in other cancer cells [33, 37]. Besides the increase in TRAIL-receptors and effects on cFLIP levels [15, 16, 37–39], particularly the Bcl-2 family members NOXA and MCL-1 accumulated in NSCLC cells after bortezomib exposure [39–41]. Cleavage of the anti-apoptotic MCL-1, which induces an imbalance between MCL-1 and NOXA, could then serve as a mechanism of bortezomib-mediated sensitization to rhTRAIL-induced apoptosis [40]. Bcl-2 was also examined in the context of bortezomib sensitization, revealing an inhibition of bortezomib and rhTRAIL-induced apoptosis in NSCLC upon Bcl-2 overexpression [16]. However, the combination of bortezomib and rhTRAIL resulted in a synergistic cytotoxic effect in Bcl-2-overexpressing T cell leukemia cells [41]. Moreover, bortezomib-mediated rhTRAIL-induced apoptosis was found to be dependent on Bak, Bik and Bim expression in prostate and colon cancer cells [42, 43]. In a previous study, we explored the apoptotic mechanisms behind TRAIL sensitivity in bortezomib-resistant NSCLC, including A549 cells. Interestingly, we found that generated bortezomib-resistant A549 cells switched to rhTRAIL sensitive phenotype. This increased sensitization to TRAIL appears to be primarily due to the relocalization of TRAIL-R1 into lipid rafts and the activation of both extrinsic and intrinsic apoptotic pathway via alterations in the expression levels of multiple Bcl-2 family members [19]. In another study, we demonstrated that TRAIL induced cell death in these NSCLC cell lines is induced via the caspase pathway. Two lines of evidence for this pathway were found, inhibition of the protection by Mcl-1 or Bcl-2 with their inhibitor obatoclax increased caspase induced apoptosis, while the pan-caspase inhibitor zVAD also inhibited TRAIL induced apoptosis [30]. This also indicates that non-apoptotic cell death, e.g., by necroptosis, is unlikely to play a role in these cells. These findings align with the broader observation that bortezomib exerts diverse effects on tumor cells, consistently tipping the balance between pro- and anti-apoptotic proteins in favor of apoptosis. While these effects may vary across different tumor types, the overall outcome remains the promotion of apoptosis.

Efficacy of clinical combinations of bortezomib and TRAIL is most likely determined by their intracellular interaction, for which each drug should show appropriate pharmacokinetics. Bortezomib showed a short initial plasma half-life of about 10 min after intravenous bolus injection [44], possibly mediated through rapid degradation by cytochrome P450 isozymes. However, bortezomib is also rapidly distributed into tissues, leading to a long elimination half-life of > 40 hours, which can be explained by subsequent release from tissues into plasma. This long tissue half-life possibly explains intracellular effects, which have been observed in clinical combination studies. For example, we observed that bortezomib selectively increased the expression of tumor cell deoxycytidine kinase, an essential enzyme in the activation of gemcitabine, leading to increased accumulation of the active metabolite of gemcitabine (gemcitabine triphosphate) in tumor cells. This activation was not observed in white blood cells, leading to a selective synergism in tumor cells [16, 45, 46].

For recombinant TRAIL plasma half-life is relatively short as well, but TRAIL shows a limited tissue penetration [47], possibly explaining its limited effect in solid tumors. Retention of TRAIL can be improved by application of a sustained release form such as pegylated TRAIL [48, 49] or a fusion protein, eftozanermin alfa, which indeed showed an increased half-life varying from 21–46 hours [50], while biomarker analysis showed induction of apoptosis in tumors from these patients. Also, a liposomal formulation of TRAIL showed improved serum pharmacokinetics, enabling bortezomib to sensitize tumor cells to TRAIL induced apoptosis in a soluble TRAIL resistant neuroblastoma model [51]. Therefore, clinical applications of TRAIL with bortezomib are likely to be active when bortezomib is administered before either liposomal or pegylated TRAIL to sensitive tumors.

TRAIL has shown synergism with several other drugs in various tumors, such as with TAS-102 [52], a drug recently approved for 3rd line colorectal cancer. Similar to the synergism observed between obatoclax, a Mcl-1/Bcl-2 inhibitor, and TRAIL [30], another Mcl-1/Bcl-2 antagonist, venetoclax, showed synergism with the TRAIL fusion protein, eftozanermin alfa, in acute myeloid leukemia cells, as well as in patients [53]. Future clinical applications of such combinations should be performed with a sustained release form.

In conclusion, we have demonstrated that bortezomib enhances rhTRAIL-induced apoptosis in NSCLC cells in a time-dependent way, involving both intrinsic and extrinsic pathways. The fact that bortezomib has the capacity to enhance rhTRAIL-induced apoptosis in NSCLC cells via multiple mechanisms strongly supports further exploration of bortezomib and TRAIL combinations as promising treatment modalities for NSCLC, warranting additional in vivo studies, which should also be performed with either a pegylated or liposomal formulation, which were beyond the scope of this study. This study focused on experimentally characterizing apoptotic signaling mechanisms in selected NSCLC cell lines. Analysis of large-scale datasets (such as DepMap or TCGA) could further elucidate how genomic and transcriptomic differences may be correlated to drug sensitivity or survival of patients. However, these databases usually do not provide drug sensitivity profiles of combinations, while the TCGA database often does not provide the type of treatment and may therefore not give information on correlations of outcome of treatment with experimental therapy, such as the response to bortezomib and rhTRAIL combination therapy. In our current research [30], we focused on mechanisms of other pro- and anti-apoptotic proteins in sensitization of NSCLC cells to rhTRAIL-induced apoptosis.

## Abbreviations

NSCLC: non-small cell lung cancer
PI: propidium iodide
TRAIL: TNF-related apoptosis-inducing ligand

## Declarations

### Availability of data and materials

The raw data that support the findings of this study are available on request from the authors (corresponding author: GJP).

### Copyright

© The Author(s) 2025.
